# Potential Hemostatic and Wound Healing Effects of Thermoresponsive Wound Dressing Gel Loaded with *Lignosus rhinocerotis* and *Punica granatum* Extracts

**DOI:** 10.3390/gels9010048

**Published:** 2023-01-06

**Authors:** Farha Yasmin Faris Taufeq, Nur Hamizah Habideen, Loageshwari Nagaswa Rao, Promit Kumar Podder, Haliza Katas

**Affiliations:** Centre for Drug Delivery Technology, Faculty of Pharmacy, Universiti Kebangsaan Malaysia, Jalan Raja Muda Abdul Aziz, Kuala Lumpur 50300, Malaysia

**Keywords:** acute wound, amphiphilic polymer, topical gel, medicinal mushroom, antibacterial dressing

## Abstract

Biologically active compounds in *Lignosus rhinocerotis* and *Punica granatum* are found to facilitate wound healing and exhibit hemostatic activity, making them a good combination as bioactives for wound dressings. This study, therefore, aimed to evaluate the potential of thermoresponsive gels loaded with *L. rhinocerotis* (HLRE) and *P. granatum* (PPE) extracts as dressings for wound treatment. The gels were prepared using Pluronic PF127 polymer and mixed with PEG 400 and/or starch prior to incorporation with both extracts (0.06 to 1 mg/mL). The gelation temperature (T_gel_) at the skin temperature was achieved when Pluronic PF127 was mixed with 22% *w*/*v* PEG 400 and reduced to 25.7 ± 0.3–26.7 ± 1.2 °C after adding HLRE and PPE. The gels exhibited satisfactory hardness (2.02 ± 0.19–6.45 ± 0.53 N), cohesiveness (0.9 ± 0.07–2.28 ± 0.4 mJ), adhesiveness (5.07 ± 2.41–19.6 ± 1.1 mJ), and viscosity (0.15 ± 0.04–0.95 ± 0.03 Pa.s), suitable for wound dressings. The optimized gels displayed high thrombin activity and cell migration rate (wound closure of 74% ± 12–89% ± 2 within 24 h), demonstrating hemostatic and healing effects. The thermoresponsive gels demonstrated advantages to be used as dressings for treating acute and open wounds.

## 1. Introduction

A wound signifies a break in the tissue’s continuity covering the body, thus permitting the entry of foreign materials into the tissues. Despite countless wound healing products being sold in the market such as semi-permeable films, hydrogels, and hydrocolloids dressings, topical thermoresponsive formulations that utilize natural extracts for fast application and healing are not available. Pluronic is well-known to possess thermoreversible properties and Pluronic PF127 is the most widely studied variant so far [1,2]. It exists as free-flowing liquid at low temperature and converts into semi-solid gel at a higher temperature, making it an ideal thermoresponsive polymer for topical drug delivery systems [3]. Thermoresponsive gels (that convert liquid to gel at skin/body temperature) are considered a good dressing for improving the self-medication of wounds owing to their ability to provide a fast-external barrier and are suitable for any wound size and site. Modification of the formulation by adding excipients such as polyethylene glycol 400 (PEG 400) and/or starch is necessary to achieve the gelation temperature (T_gel_) that is suitable for topical applications. PEG 400 is a water-soluble polymer and used to increase T_gel_ by forming stable micelle clusters with Pluronic PF127. Starch is also commonly used to alter T_gel_ due to its ability to form inclusion complexes with hydrophobic compounds. Generally, thermoresponsive topical drug delivery systems are advantageous with respect to gel spreadability on different sizes and locations of wounds as they flow on the skin upon application. In addition, it allows the gels to be easily distributed and active ingredients to be released [4].

The use of traditional medicine or natural compounds has been of great interest in the pharmaceutical field for many years including the development of wound healing agents [5,6]. For example, herbal extracts have been investigated for their healing properties and developed as advanced wound dressings [7]. *Lignosus rhinocerotis* is a medicinal mushroom and traditionally used for wound treatment [8] by indigenous communities in Malaysia. Its sclerotium holds various medicinal values including anti-microbial and antiviral activities [9,10,11,12,13], making it a good candidate as a natural wound healing agent. Traditionally, people cut the sclerotium of the mushroom into pieces and boil in water prior to its application for medicinal purposes [14]. Therefore, hot water extraction was applied in this study to resemble the traditional preparation of *L. rhinocerotis*. 

In addition to that, the wound dressing should provide a hemostatic effect to avoid excessive bleeding. In addition to high antioxidant and anti-inflammatory properties [15], the peel extract of *Punica granatum* (pomegranate) exhibits hemostatic activity owing to its high polyphenols content such as ellagitannins [16]. The wound healing potential of *Punica granatum* was also associated with a high polyphenols content [17,18]. Therefore, the combination of both extracts in a wound dressing is anticipated to provide synergistic effects to allow faster healing of acute wounds. Moreover, none utilize the combination of Pluronic PF127 and both extracts that offers hemostatic and healing properties that may be useful for sport-related wounds that are urgently needed in facilitating the speedy recovery of injured athletes [19]. Several studies have been carried out for increasing the rate of wound healing and hemostatic effects [20,21]. However, a new class of wound dressing is still required for achieving better outcomes by fulfilling other needs such as the prevention of infections and easy application on the affected areas [22]. Other than offering multiple actions for speedy wound healing, the combination of thermoresponsive gels and both extracts is expected to allow self-medication.

In the current study, extracts of *L. rhinocerotis* (HLRE) and *P. granatum* (PPE) were incorporated into topical thermoresponsive gel formulations of Pluronic PF127. Phytochemical screening of both extracts was first carried out using Gas Chromatography-Mass Spectrophotometry (GC-MS) to identify potential compounds that might contribute to the wound healing effects. Later, all the gels were evaluated for various properties including T_gel_, texture, rheology, and morphology as well as biological effects (antibacterial and thrombin activities). Wound healing effects of the gels were also evaluated via the in vitro cell migration assay (wound scratch assay).

## 2. Results and Discussion

The optimal formulation of thermoresponsive gels was selected based on T_gel_ and the texture profile of the resulting gels. The optimized gels were later incorporated with the natural extracts, HLRE and PPE, followed by physicochemical characterization as well as biological evaluation including antibacterial, thrombin, and wound healing activities. In this study, HLRE of 0.125 mg/mL was used for the development of thermoresponsive gels. The concentration was selected as the highest proliferative activity was observed as determined by a cell migration assay previously (data are not shown). Moreover, no sign of cytotoxicity was detected for HLRE up to 0.25 mg/mL, as the percent of cell viability was above 80%. The blank gel (F5) and those loaded with HLRE also did not show any cytotoxicity effect, as the percent of cell viability was 90% and 82%, respectively (Supplementary Figure S1).

### 2.1. Phytochemical Screening of HLRE and PPE

Compounds in HLRE and PPE were identified qualitatively based on their retention times and mass spectral fragmentation patterns. For HLRE, more than 40 compounds were identified, in which 13 compounds (in bold) were found to be associated with wound healing properties (Table 1 and Supplementary Figure S2A). Previously, HLRE was reported to contain oligosaccharides, polysaccharides, fatty acids, and phenols as determined by LC-MS [23]. On the other hand, the qualitative GC-MS analysis identified 80 compounds in PPE, which represents 100% of the extract compositions. The major compounds identified include heptadecane, 9-octyl- (alkanes, 12.69%), pentatriacontane (alkanes, 10.99%), heneicosane (alkanes, 9.53%), pentatriacontane (alkanes, 5.51%), 5-hydroxymethyfurfural (aldehyde, 5.451%), 4H-Pyran-4-one, 2,3-dihydro-3,5-dihydroxy-6-methyl- (flavonoid, 3.94%), oxalic acid, allyl octadecyl ester (ester, 3.64%), octatriacontyl pentafluoropropionate (carboxylic, 2.72%), and ethanol 2-(octadecyloxy)(primary alcohol, 2.504%). Other compounds detected were in trace amounts (less than 2.5%) (Table 2 and Supplementary Figure S2A). Most of the compounds identified are reported to exert antioxidant activities. Antioxidants are useful in wound healing owing to their ability in controlling wound oxidative stress by regulating damages occurring on biological molecules (DNA, protein, lipids, and body tissues), resulting in accelerated wound healing [24].

### 2.2. T_gel_

The T_gel_ of all the gel formulations is shown in Table 3. Pluronic PF127 gel (F1) made of 25% *w*/*v* of the polymer turned into gel at below 25 °C, demonstrating that Pluronic PF127 alone was not suitable to be developed as a thermoresponsive gel. The difficulties in manufacturing, handling, and administration may arise if the T_gel_ is below 25 °C as the gel is formed at room temperature [25]. Moreover, the target T_gel_ for blank gels should be high enough as it may be reduced to the value lower than the acceptable range (below 25 °C) after loading with active ingredients due to the disturbance of micelle formation of the polymer.

**Figure 1:**
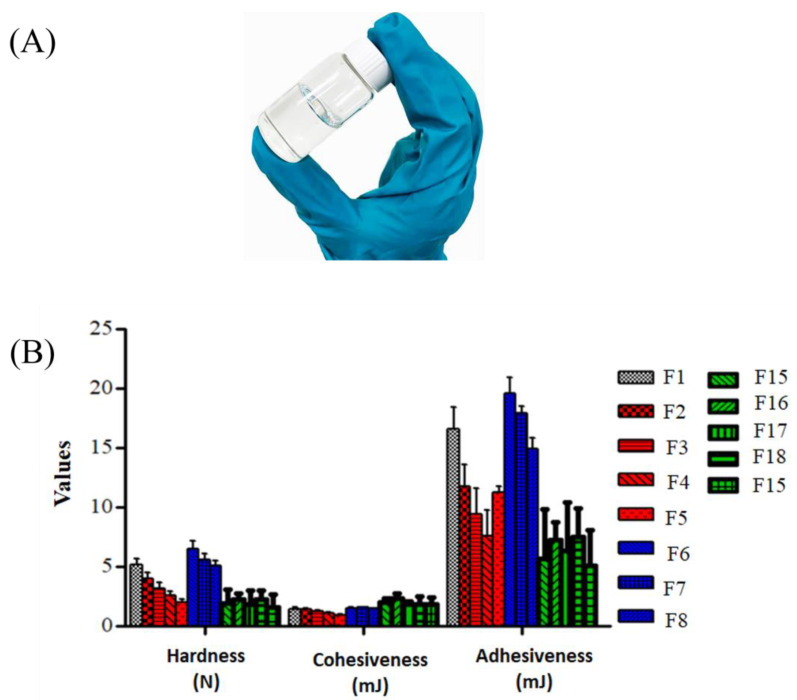
(**A**) Physical appearance of Pluronic PF127 gel at ambient temperature. (**B**) Texture profile analysis (TPA) of thermoresponsive gels of different formulations and containing natural extracts, n = 3.

In an attempt to increase T_gel_, different PEG 400 concentrations were added into the formulation (F2-F5). The T_gel_ of the resulting gels increased from 27.3 ± 0.6 °C to 37.0 ± 1.0 °C with the increased PEG concentration from 5% to 22% *w*/*w* (*p* < 0.05, one-way ANOVA, Bonferroni’s post hoc analysis). PEG 400 modifies the micelles of Pluronic PF127 through the binding of the ester group from PEG 400 to hydrophilic chains of Pluronic PF127, promoting the dehydration of the hydrophobic polyoxypropylene (PPO) block and, thus, causing an increase in the entanglement of the adjacent micelles. On the other hand, for formulations with the addition of starch (F6–F8), T_gel_ for all the concentrations was lower than the Pluronic PF127 gel (F1) (22.3 ± 0.6 °C to 24.3 ± 0.6 °C). The decrease in T_gel_ was most probably due to the disturbance of micelle packing and entanglement of Pluronic PF127 [26] in the presence of starch. PEG 400 and starch (F9–F14) were also combined and added into the formulation, but the results did not show any advantage for achieving the target T_gel_. Hence, the formulations were not proceeded further. A higher T_gel_ than 37 °C should also be avoided as the gel remains liquid at body temperature and, thus, the formulation will neither provide a solid artificial barrier nor act as a sustained release depot [27,28]. Therefore, F5 (with the addition of 22% PEG 400) was selected for further analysis and used to incorporate HLRE and PPE, to produce gels loaded with natural extracts (F15–F19). As expected, the incorporation of HRLE and PPE (F15–F19) reduced the T_gel_ (25.7 ± 0.3–26.7 ± 1.2 ℃) (Table 3), probably due to the disturbance of micelle formation. Despite the reduced T_gel_, the values were above 25 °C. The critical micelle temperature of gelation should be between room and body temperature [29], and as the wound temperature could be lower than the body temperature (31–34 °C) [30], this formulation could still be further developed as a thermoresponsive gel for topical applications. Moreover, the formulation could be kept at a lower temperature before applying on any acute wound, offering various advantages from local cooling such as a hemostatic effect and protection of injured tissues due to an increased level of hemoglobin oxygen saturation in both superficial and deep layers [31]. The addition of both extracts did not influence the general behavior of the gels as they exhibited in a reversible thermal gelation. As shown in Figure 1A, the gel remained liquid in the cold condition (sol phase) and turned into a semisolid (gel phase) at higher temperature (25–37 °C). The thermosensitive nature of the gel is contributed by its components that are made up of repeating blocks of polymer consisting of polyethylene oxide (PEO) and polypropylene oxide (PPO). The hydrophobic segment PPO is located at the core and surrounded by the hydrophilic segment of PEO [32,33]. Taken together, the T_gel_ of the thermoresponsive gels developed in this study is considered suitable for wound drug delivery. 

### 2.3. TPA 

The hardness, adhesiveness, and cohesiveness of thermoresponsive gels are presented in Figure 1B. Hardness is an important parameter to indicate gel applicability at the administration site, whereas cohesiveness is useful to predict the spreadability of the gel as well as its removal from the container. A low value of hardness and cohesiveness is desirable for easy removal of the product from the container and administration on the skin [34]. In general, the hardness and cohesiveness reduced with increasing PEG 400 or starch concentrations. The higher values of hardness and cohesiveness for F6–F8 (starch) than that of F2–F5 (PEG) suggested that the mechanical properties of F2-F5 were more suitable for topical applications. 

Adhesiveness indicates the adhesion of gel on skin that will also affect the drug retention time at the site of action [35]. Despite the adhesiveness of F5 not being the highest, it was selected for further development owing to other desirable properties (low hardness and cohesiveness with T_gel_ that is close to skin/body temperature). For the formulations containing HLRE and PPE (F15-F19), the hardness was lower than that of F5 (the optimal formulation consisting of Pluronic PF127 and 22% *w*/*v* PEG 400 and unloaded/blank gel) (*p* < 0.05, two-way ANOVA, Bonferroni’s post hoc analysis), but PPE concentration had no influence on the hardness. Contrarily, the cohesiveness of F15–F19 was comparable to that of F5 and it was affected by the PPE concentration, as it increased with higher PPE concentrations. The findings indicated that the addition of natural extracts did not influence the gel cohesiveness but affected the hardness and adhesiveness. The findings also suggested easy handling of the resulting gels containing HLRE and PPE owing to their low hardness and cohesiveness as well as acceptable values of adhesiveness. Despite no precise value being available that can be used as a standard reference for adhesiveness, it is generally accepted that products with high adhesive properties are better suited for topical administration to prolong their time at the application site [35]. The gels may be utilized further owing to their high adhesiveness, and this could be linked to the high polymer employed in the gel preparation [34,36].

### 2.4. Rheology 

**Figure 2:**
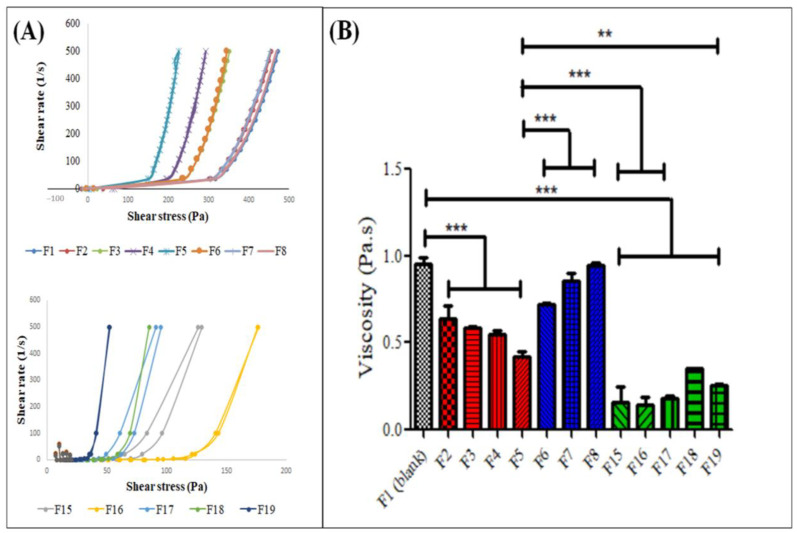
(**A**) Rheological profile and (**B**) viscosity of thermoresponsive gels of different formulations and containing the natural extracts at 25 °C, n = 3. ** and *** indicate significant differences at *p* < 0.05 and 0.01, respectively.

Rheological characteristics such as viscosity identify the flow and deformation of products, the important data for production, filling, packaging, and storage during manufacturing. Figure 2A depicts shear thinning curves for the gels examined (F1–F8 and F15–F19), which have pseudo-plastic or non-Newtonian properties due to their thermosensitive nature. Their viscosity decreased when the shear rate was increased as the alignment of the polymer molecules was disrupted under the application of the shear stress, causing the molecules to flow easily with a gradual decrease in viscosity [37]. This also suggested that the thermoresponsive gels were able to flow readily.

Figure 2B shows the apparent viscosity of thermoresponsive gels at 500 s^−1^. F5 had the lowest viscosity as compared to F1 and other formulations (F2-F8) (*p* < 0.05, one-way ANOVA, Bonferroni’s post hoc analysis). This could be due to the disruption of the polymeric structure of the tri-blocks, which affects the micelle formation [38]. This finding further supported the selection of F5 as a topical vehicle for HLRE and PPE (F15–F19) as any formulation with high viscosity tends to have trouble in handling and administration at the administration site [39]. The viscosity further reduced when HLRE and PPE were added into the formulation, suggesting that the addition of more substances can probably further disrupt the micelles of Pluronic PF127. 

### 2.5. ATR-FTIR Analysis

**Figure 3:**
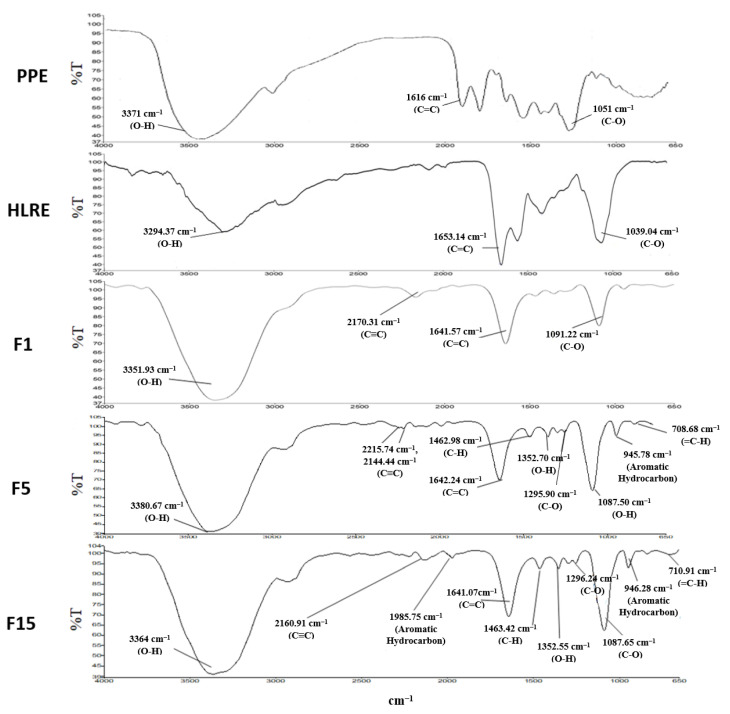
FTIR of PPE, HLRE, F1, F5, and F15.

In this analysis, the spectrum of blank and gels containing HLRE and PPE was compared to identify the interactions among the active ingredients, the polymer, and other excipients (Figure 3). Blank Pluronic PF127 gel showed absorption peaks at 3351.93 cm^−1^ (O-H Stretching), 2170.31 cm^−1^ (C≡C), 1641.57 cm^−1^ (Alkene C=C), 1091.22 cm^−1^ (C-O Stretching), and 946.43 cm^−1^ (=C-H Bending). In the spectrum of PEG 400, additional peaks were observed at 2144.44 cm^−1^ (C≡C) and 708.68 cm^−1^ (=C-H Bending). Sclerotia of *L. rhinocerotis* extract contain proteins, polysaccharides, and/or polysaccharide–protein complexes [40]. The peaks for the thermoresponsive gel containing HLRE were observed at 3365.73 cm^−1^ (O-H Stretching), 2222.59 cm^−1^ (C≡C), 2134.91 cm^−1^ (C≡C), 1974.27 cm^−1^ (aromatic hydrocarbon (overtone)), 1643.26 cm^−1^ (C=C), 1462.81 cm^−1^ (C-H), 1352.67 cm^−1^ (O-H Bending), 1295.68 cm^−1^ (C-O Stretching), 1087.09 cm^−1^ (C-O Stretching), and 945.63 cm^−1^ (=C-H Bending). These spectra are similar to another study [18]. The peaks observed in the blank gel appeared in the spectrum of gel containing HLRE, indicating chemical compatibility of the active ingredient, the polymer, and excipients. With the addition of PPE, the spectrum showed major peaks of 3364.00 cm^−1^ (O-H Stretching), 1641.07 cm^−1^ (C=C), 1296.24 cm^−1^ (C-O Stretching), and 1087.65 cm^−1^ (C-O Stretching). These peaks correspond to a previous study [41]. The peaks of blank gel could still be observed after incorporating HLRE and PPE, suggesting that the constituents added into the gel formulation were compatible with no signs of interactions between the polymer and the extracts. 

### 2.6. Morphology

**Figure 4:**
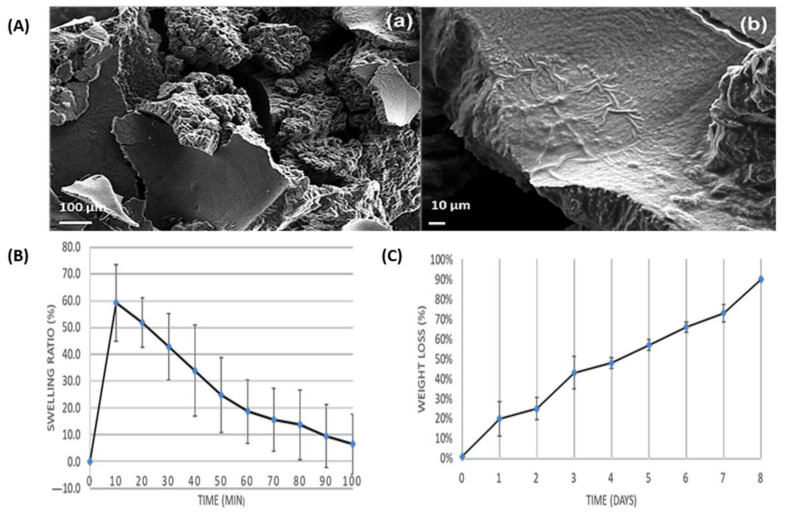
(**A**) FESEM micrographs of thermoresponsive gel containing HLRE and PPE (F15) at 100 kX (**a**) and 200 kX (**b**). (**B**) Swelling ratio and (**C**) biodegradation of thermoresponsive gels containing HLRE and PPE (F15), n = 3.

The thermoresponsive gels containing natural extracts of HLRE and PPE appeared as a transparent and viscous semisolid at 37 °C. Based on the three-dimensional (3D) topographic FESEM micrographs (Figure 4), the F15 gel had a combination of smooth and rough surfaces. A similar morphology was observed for other gels containing HLRE and PPE. Smoother surfaces have been associated with a high degree of phase transition into gel [42]. As the gel was combined with several other constituents (PEG 400, HLRE, and PPE), it could be a factor for the formation of rough surfaces on certain parts of the gel. Furthermore, the thickness of the gels was estimated as approximately 107 ± 23 µm, based on the measurement of the image in Figure 4Ab.

### 2.7. Swelling Properties and Biodegradation 

At the beginning, the thermoresponsive gels swelled rapidly to the maximum degree (59.2% ± 14.3) within 10 min due to the absorption of solvent (PBS). After that, the swelling started to reduce slowly, probably as a result of dilution on the gels (Figure 4B). For degradation analysis, the thermoresponsive gels were slowly degraded within 8 days (Figure 4C). Weight loss was observed over time as the percent of weight loss increased to 20 ± 8% within a day and continued to increase to 90 ±10% after 8 days of incubation. The findings, therefore, suggested that the thermoresponsive gel can absorb fluids to a certain extent. Owing to the Pluronic PF127 components that formed self-assembling micelles in the gel structure, the degradation and mass erosion of the micelles occurred due to the hydrolytic scission of cleavable ester linkage between the Pluronic PF127 and acrylate group upon exposure to an aqueous environment of a higher temperature (37 °C), causing the Pluronic PF127 polymer chains to readily undergo hydrolysis [43].

### 2.8. Antibacterial Activity

HLRE has no antibacterial activity as determined previously by our group [23,44]. Therefore, in the current study, only PPE was tested for antibacterial activity against *Staphylococcus aureus,* the most common wound infecting bacteria. Only the high concentrations of PPE (0.5 and 1 mg/mL) exhibited antibacterial activity against *Staphylococcus aureus*. However, the activity was almost three-fold lower than the positive control. Tannins found in PPE may contribute to the antibacterial activity [45,46]. Moreover, the thermoresponsive gels containing natural extracts did not show any antibacterial activity, probably due to the low antibacterial activity of PPE, as well as the slow release of PPE from the gels (Table 4).

### 2.9. Thrombin Activity

**Figure 5:**
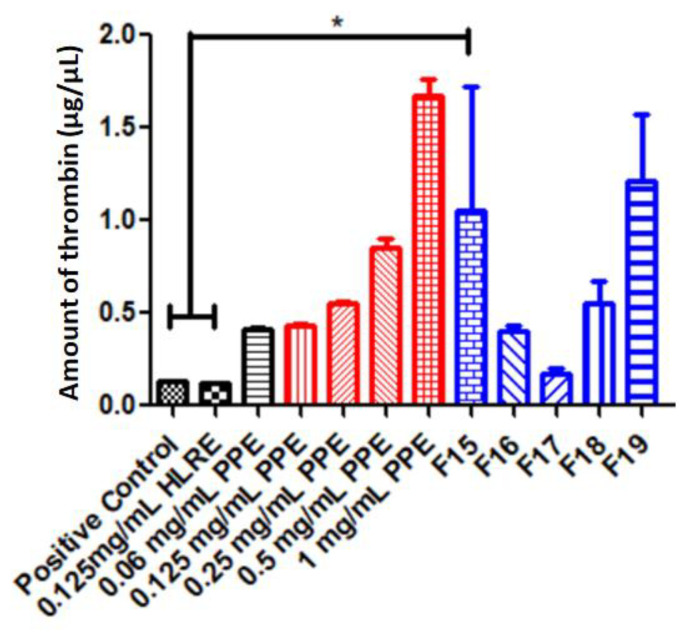
Thrombin activity of HLRE, PPE, and the gels containing both extracts (with different PPE concentrations), n = 3. * indicates significant difference at *p* < 0.05.

Thrombin is an enzyme involved in the final coagulation cascade, to form fibrin clots [47]. Thrombin activity is measured to determine the hemostatic effects (stop bleeding) of a formulation. Both HLRE and PPE were reported to exhibit a hemostatic effect owing to the presence of biological active compounds such as protease [48] in HLRE, while tannins [20] and ellagic acid were in PPE [49]. Figure 5 shows the thrombin activity for HLRE and PPE as well as the responsive gels containing both extracts. All samples have been shown to exhibit thrombin activity, and the highest activity was recorded for the highest PPE concentration (1 mg/mL), significantly higher than the positive control and HLRE (*p* < 0.05, one-way ANOVA). The thrombin activity of PPE was concentration-dependent, as the higher the PPE concentration, the greater thrombin activity was recorded. When incorporated into the thermoresponsive gel, the thrombin activity was generally reduced except for the lower concentrations of PPE (0.06 and 0.155 mg/mL of PPE). The finding, therefore, shows the possibility of interaction between HLRE and PPE at higher concentrations (above 0.25 mg/mL) with the polymer and/or excipients, thus limiting the thrombin activity. Biomaterials such as Pluronic may trigger protein adsorption and subsequent blood clot formation [50], providing synergistic effect when combined with both extracts. However, the hemostatic effect was enhanced for the lower PPE concentrations only (0.06 and 0.125 mg/mL), indicating that PPE concentration had more impact on the hemostatic activity than the intrinsic property of the gel.

### 2.10. In Vitro Wound Healing Potential

**Figure 6:**
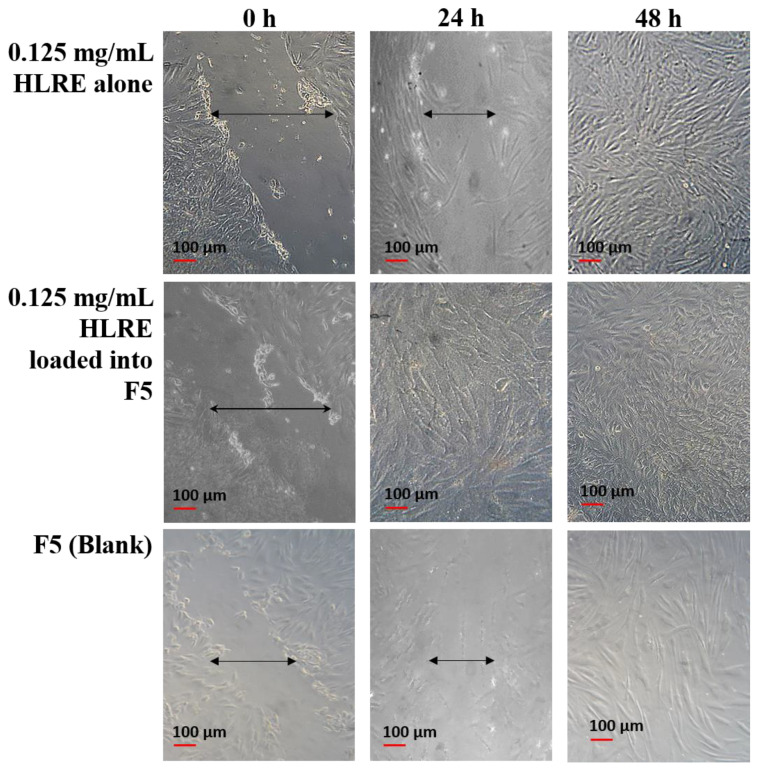
Cell migration for HLRE and thermoresponsive gel (F5) containing HLRE.

The wound scratch assay is a well-developed technique to study cell migration from the wound edges into the cell-free area [51]. The analysis was first carried out for HLRE and its thermoresponsive gel (F5 containing HLRE) for evaluating the effect of the gel on the healing property of HLRE, one of the main properties needed in wound treatment. Based on the results (Figure 6), the scratch area achieved a full confluence within 48 h for HLRE. Genomic features of *L. rhinocerotis* extract revealed the presence of proteins encoded for cell division, replication, and repair, thus proving the uniqueness of *L. rhinocerotis* as a wound healing agent [52]. The incorporation of HLRE into the thermoresponsive gel (F5) had enhanced the cell migration as the confluence of the scratch area was fully achieved within 24 h, showing that the gel also accelerated cell migration. Despite the blank gel having the slowest rate of migration, the activity of PEG could also contribute to the healing promotion through the polymer coating or siliconization of the injured surface [53] as a higher rate of migration was observed when HLRE was incorporated into the gel. 

**Figure 7:**
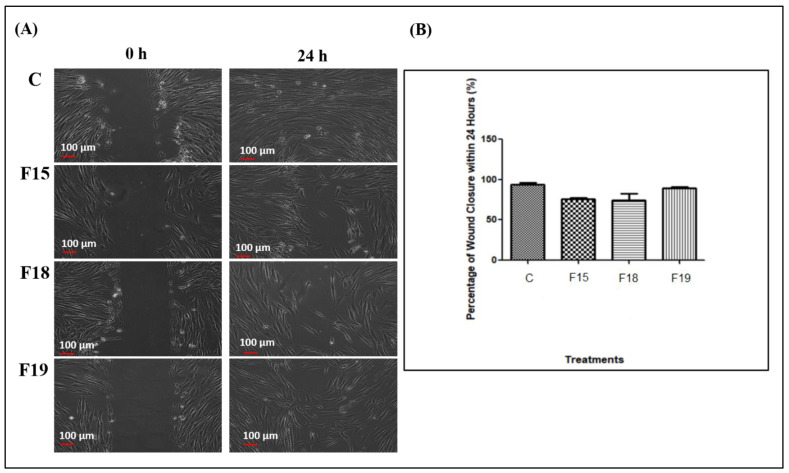
(**A**) Wound scratch assay for HDFs treated with DMEM only as an experimental control C, F15, F18, and F19. (**B**) Percent of wound closure within 24 h for HDFs treated with different treatments.

Later, the wound scratch assay was carried out for the thermoresponsive gels containing HLRE and PPE of different concentrations. For this study, only those that displayed high thrombin activity (F15, F18, and F19) were tested as thrombin is important in the wound healing process too in addition to providing a hemostatic effect. Thrombin plays a key role in the early inflammatory phase, stimulating chemotaxis and production of inflammatory cytokines and chemokines [54]. Production of thrombin by the coagulation cascade also provides a long-lasting hormone-like effect on fibroblast proliferation that is important for wound healing [55,56,57]. As shown in Figure 7, the percent of wound closure for HDFs treated with F19, F15, and F18 was 89.3% ± 2.4, 76.0% ± 1.5, and 74.4% ± 11.5, respectively, which is comparable to DMEM only (positive control, 94.1% ± 2.4) (*p* > 0.05 one-way ANOVA, post hoc Bonferroni) after 24 h. All samples achieved 100% wound closure after 48 h (data not shown). The migration rate of the gels was comparable to the control, showing the normal proliferation and migration rate of cells treated with the gels. Owing to the gel properties that provide other benefits including a physical barrier to external contaminants and hemostatic effects, the gel is expected to promote wound healing in vivo. The results also demonstrated that high thrombin levels in F15, F18, and F19 contributed to the wound healing efficacy of the gels. The presence of thrombin provides beneficial effects on the cell activities and tissue regeneration [58]. The use of Pluronic PF127 gels as a vehicle also facilitates wound healing efficacy. Pluronic PF127, a thermoreversible polymer, possesses wound healing properties and is used as a vehicle for several routes of drug administration including oral and topical [59]. The gels were also incorporated with other polymers to achieve suitable strength, viscosity, and T_gel_ [60]. The finding demonstrated that the cell migration of the three gels was comparable to the normal healing and could be applied as wound dressings with healing properties.

## 3. Conclusions

In this study, topical thermoresponsive gels based on Pluronic PF127 polymer were developed as a wound dressing and vehicle for HLRE and PPE. HLRE and PPE are the natural extracts used traditionally for wound healing and are known to contain bioactive compounds with hemostatic and wound healing properties. Although the thermoresponsive gels loaded with HLRE and PPE did not show antibacterial activity, they exhibited a very high hemostatic effect. The in vitro wound healing efficacy study through the wound scratch assay also demonstrated that the gels promoted the wound healing as they showed the same healing rate to the normal healing. Taken together, the thermoresponsive gels containing HLRE and PPE have shown some potential to be used as a wound dressing with hemostatic and healing properties for treating acute wounds. However, further modification and improvement are needed to provide a more natural and holistic option for wound treatment with fewer side-effects.

## 4. Materials and Methods

### 4.1. Materials

*L. rhinocerotis* (Tiger Milk Mushroom) sclerotia powder was obtained as a gift from Lignas Bio Synergy Plt., Selangor, Malaysia). *P. granatum* fruits were purchased from a supplier in Kuala Lumpur, Malaysia. Pluronic F-127 was purchased from Sigma-Aldrich (Missouri, USA). PEG 400 with an average molecular mass of 380–420 g/mol was procured from Merck (Darmstadt, Germany). For the antibacterial test, *Staphylococcus aureus* (ATCC 6538) was obtained from the Microbiology Laboratory of Faculty Pharmacy, Universiti Kebangsaan Malaysia, Kuala Lumpur. Mueller–Hinton broth (MHB) and Mueller–Hinton agar (MHA) were purchased from the Difco laboratory of Becton Dickinson Company, USA. Gentamicin sulphate was purchased from Nascalai Tesque, Kyoto, Japan. SensoLyte^®^ 520 Thrombin Assay Kit *Fluorometric* was purchased from AnaSpec (California, USA). Human dermal fibroblasts (HDFs, passage 2–8) were used for cell migration assays. Dulbecco’s modified Eagle’s medium (DMEM) (high-glucose) comprising 4.5 g/L of D-(+)-Glucose, L-Glutamine, phenol red, HEPES, and sodium pyruvate was obtained from Nacalai Tesque (Kyoto, Japan). A solution of 0.05% Trypsin/0.53 mM EDTA was also procured from Nacalai Tesque (Kyoto, Japan). Pen-Strep (penicillin streptomycin) and fetal bovine serum (FBS) were purchased from Gibco (USA), while phosphate-buffered saline (PBS) was obtained from Invitrogen (USA).

### 4.2. Preparation of HLRE

Hot water extraction was used to extract sclerotium *L. rhinocerotis.* The mass-to-volume ratio of 1:20 (g/mL) was used by which 100 g of *L. rhinocerotis* sclerotia powder was soaked in 2 L of distilled water. The mixture was subjected to hot water extraction by using a Soxhlet extractor (Merck, Darmstadt, Germany) for 60 min at 95–100 °C. The mixture was then filtered using Whatman no. 1 filter paper to obtain HLRE. HLRE was kept overnight at −80 °C prior to freeze-drying using a ScanVac CoolSafe Freeze-Dryer (Lynge, Denmark) at −100 °C. Dark brown and fluffy powder was obtained for HLRE.

### 4.3. Preparation of PPE

*P. granatum* peels were cut into small pieces prior to drying in an oven at 40 °C for 48 h. The dried peels were grinded using a mechanical grinder followed by sieving to obtain fine powder. *P. granatum* peel powder was extracted using hot water at a mass-to-volume ratio of 1:6, following a previous method reported elsewhere [17,23,48,61] with a slight modification. An amount of 50 g of *P. granatum* peel powder was soaked into 300 mL of distilled water. The mixture was extracted using a Soxhlet extractor at 100 °C for 2 h. The mixture was let to cool prior to filtering using Whatmann No 1 filter paper. The extract was kept in a freezer at −80 °C overnight. The extract was then freeze-dried using a ScanVac CoolSafe Freeze-Dryer (Lynge, Denmark). Dark brown and fluffy powder was obtained for PPE.

### 4.4. Phytochemical Screening of HLRE and PPE Using GC-MS

GC-MS analysis was performed using a GC Agilent 7890A gas chromatograph (GC) (Agilent Technologies, Inc., Santa Clara, CA, USA), directly coupled to the mass spectrometer system (MS) of an Agilent 5975C inert Mass-Selective Detector with a triple-axis detector. It was equipped with a polar Agilent HP-5 MS (5% phenyl methylpolysiloxane) capillary column (30 m × 0.25 mm in diameter and 0.25 μm in film thickness) that was increased to 300 °C and held for 10 min following an initial temperature at 70 °C. Helium was used as a carrier gas with a linear velocity of 1 mL/min. The MSD Chemstation was used to identify all the peaks in the raw GC chromatogram. A library search was carried out for all the peaks using NIST/EPA/NIH version 2.0. The results were combined in a single peak table.

### 4.5. Preparation of Thermoresponsive Gels

The thermoresponsive gels (25% *w*/*w*) were prepared using a cold method that was established in our laboratory [4]. Pluronic PF127 (12.5 g) was slowly added into 50 mL of cold distilled water under a continuous magnetic stirring (Daihan Scientific WiseStir, Korea) at 200 rpm for 4 h. Afterward, the mixture was kept at 4 °C overnight. The next day, using the same cold method, different concentrations of PEG 400 (5, 10, 15, and 22% *w*/*v*) were added into the mixture and stirred continuously at 300 rpm for 4 h. The same steps were repeated for different concentrations of starch (500, 1000, and 1500 mg/L) and the combination of PEG and starch.

### 4.6. Incorporation of HLRE and PPE 

A solution of HLRE (0.125 mg/mL) was mixed with different concentrations of PPE (0.06, 0.125, 0.25, 0.5, and 1 mg/mL). Later, the mixture was freeze-dried using a ScanVac CoolSafe Freeze-Dryer (Lynge, Denmark) before the powder was loaded into the optimized formulation of Pluronic PF127 gel through the cold method (by placing the mixture on an ice bath). The mixture was stirred for 4 h at 4 °C before being kept in a 4–8 °C refrigerator until further analyzed. 

### 4.7. T_gel_ Measurement

In a beaker, a cold sample solution (4 °C) with a magnetic stirrer was placed in the preheated evaporating dish containing water. The temperature was maintained at 100 °C under constant magnetic stirring at 200 rpm. A thermometer was immersed in the cold sample solution to monitor the temperature. The temperature where the magnetic stirrer stopped the stirring was recorded as T_gel_ [4]. Triplicate measurement was carried out for each sample.

### 4.8. Texture Profile Analysis (TPA)

The texture profile of the resulting thermoresponsive gels was analyzed using a Brookfield CT-3 Texture Analyzer (USA) operating in compression mode. The parameters were set at 0.1 N and 1 mm/s for trigger load and test speed, respectively, as well as a holding time of 0.15 s. Each gel (10 mL) was placed in a glass jar with an analytical probe (with 38.1 mm diameter disc) inserted into the jar during each analysis. The gel hardness, adhesiveness, and cohesiveness were obtained from the generated graph using TexturePro CT V1.5 Build 20 software (Middleboro, MA, USA). The test was repeated three times for each sample.

### 4.9. Rheological Analysis

Rheological flow properties of the thermoresponsive gels at 25 ± 0.1 °C were analyzed using a Bohlin Gemini Rheometer (Worcestershire, United Kingdom). A cone-and-plate geometry with a 20 mm diameter and 2° angle system was used and set to operate in continuous shear analysis mode. The upward and downward flow curves for each gel were measured over the shear rate ranging from 0 to 500 s^−1^ with the application time of 3 min. The rheological flow curves were determined by plotting viscosity (Pa.s) against shear rate (s^−1^) and shear stress (Pa) against shear rate (s^−1^) using the Bohlin Software: GEMINI 200 (Westborough, MA, USA). Three independent measurements were taken for each gel. The average apparent viscosity of each sample was obtained at 500 s^−1^.

### 4.10. FTIR Spectroscopic Analysis

FTIR spectroscopy (PerkinElmer^®^ Spectrum 100 FT-IR spectrometer, PerkinElmer, MA, USA) was used to analyze natural extracts, blank gels (Pluronic PF127 gel (F1)), and the combination of Pluronic PF127 and 22% *w*/*v* PEG 400 (F5) and gels containing natural extracts (HLRE and the combination of HLRE and PPE). The samples were prepared using the potassium bromide (KBr) pellet method prior to placing onto the holder of the sample compartment. The analysis was carried out for 16 scans at 4 cm^−1^ resolution within a range of 4000 to 650 cm^−1^. 

### 4.11. Morphological Analysis

Morphological analysis of the thermoresponsive gels containing natural extracts (HLRE and PPE) was performed using a Field Emission Scanning Electron Microscope FESEM (Merlin Compact, Zeiss, Magnification 12-2000 kX). Samples were freeze-dried prior to viewing under the microscope. The images were captured at three different locations of the gels surface and at the magnification of 100 and 500 kX.

### 4.12. Swelling Analysis

The thermoresponsive gel was freeze-dried prior to cutting into three pieces. The gel pieces were then measured for their weight before soaking in 2 mL of PBS solution (pH 7.4). The weight was recorded at predetermined time points until 100 min. At each time point, PBS was removed and the remaining PBS on the gel surface was eliminated using a filter paper. The weight of swollen gel was measured, and the swelling degree (SD) was calculated using the following formula:

$$\mathrm{SD}=\frac{\mathrm{Ws}-\mathrm{Wd}}{\mathrm{Wd}}\times 100\%$$

where SD is swelling degree, Ws is the swollen weight, and Wd is the initial weight of dried gel.

### 4.13. Biodegradation Analysis

For biodegradation analysis, the thermoresponsive gels were incubated in a micro-tube containing 1 mL of culture medium and placed in a thermostatic bath unit at 37 °C. At predetermined time points, weight loss of the gels was determined by centrifuging the samples at 10,000 rpm for 5 min. After that, the samples were dried and weighed. Biodegradation (%) was calculated using the following formula:

$$Degradation\;\left(\%\right)=\frac{d0-\mathrm{dt}}{d0}\times 100\%$$

where d0 is the initial weight (before degradation) and dt is the weight at a predetermined time point (after degradation).

### 4.14. Determination of Antibacterial Activity 

An antibacterial assay was carried out using the agar well diffusion method as reported previously [44]. Gram-positive bacteria *Staphylococcus aureus* (ATCC 6538) was selected for this experiment as it is the most common wound infecting bacteria. Three colonies of the bacterium were inoculated into 5 mL of MHB and incubated for 24 h. The turbidity of the inoculum was adjusted to the absorbance of 0.08–0.1 using a UV-Vis spectrophotometer (Shimadzu 1800, Japan) at 600 nm to obtain a standard bacteria suspension (1–2 × 10^8^ CFU/mL). A sterile cotton swab was dipped into the bacteria suspension and swabbed on the MHA plate. Each sample (100 µL) was loaded into the MHA holes. Gentamicin 0.1% and distilled water was used as the positive and negative control, respectively. The MHA plates were incubated for 24 h at 37 °C prior to measuring the zone of inhibition.

### 4.15. Thrombin Activity Assay

A commercial kit, SensoLyte^®^ 520 kit Thrombin Assay Kit *Fluorometric* (AnaSpec, Fremont, CA, USA) was used to detect the enzymatic activity of thrombin. A volume of 50 µL of standard samples containing 5 nM thrombin (as experimental control) and tested samples were added into a 96-well plate. An equal volume of diluted thrombin substrate solution was added into the well. The mixture was mixed by shaking the plate gently for 30 s and incubated for 60 min at 37 °C in an incubator. Afterward, 50 µL of stop solution was added into the well before measuring the fluorescence intensity at Ex/Em = 490 nm using a microplate reader (Bio Tek PowerWave XS, Winooski, VT, USA).

### 4.16. Wound Scratch Assay

A cell migration assay was carried out as previously reported [44,62]. In this assay, 5 × 10^4^ cells/well of HDFs in DMEM supplemented with 10% FBS and 1% Pen-Strep solutions were seeded in a 24-well plate and incubated at 37 ℃ in an incubator with 5% CO_2_. Once confluent, the cells were washed with PBS solution prior to creating a scratch using a micropipette tip (10 µL) in the middle of each well. The cells were washed with DMEM to remove cell debris after scratching. The sample (sample-to-growth-media-volume ratio of 1:1) was then slowly added into the well and incubated for 24 and 48 h. The migration of cells in each well was visualized by using an inverted phase contrast microscope (Olympus CK30, Japan) connected to a digital camera (Xcam-α) composed of DigiAcquis version 2.0 software. A series of points were set and the pictures within the area of the set point were captured at 0, 24, and 48 h. The percentage of wound closure was calculated as follows:

$$wound\;closure\;\left(\%\right)=\frac{w\left(0\right)-w\left(t\right)}{w\left(0\right)}\times 100$$

where w(0) is the wound area at time 0 and w(t) is the wound area at specific time.

### 4.17. Statistical Analysis

All data are presented as mean ± SD. The data obtained were further analyzed using one-way ANOVA or two-way ANOVA followed by a post hoc Bonferroni test using GraphPad Prism software (5th Version). Values of *p* < 0.05 indicate statistical significance among the tested groups. 

## Figures and Tables

**Table 1:** Chemical compounds identified in HLRE via GC-MS analysis.

Peak Number	Retention Time	Area (%)	Identified Compounds
1	8.014	0.364	Trimethylsilyl fluoride
2	**8.161**	**0.482**	**l-Alanine, N-(trimethylsilyl)-, trimethylsilyl ester**
3	**9.677**	**0.085**	**Ethanedioic acid, bis(trimethylsilyl) ester**
4	**10.393**	**0.249**	**l-Leucine, trimethylsilyl ester**
5	10.778	0.093	1,1,1,3,5,5,7,7,7-Nonamethyl-3- (trimethylsiloxy)tetrasiloxane
6	**11.105**	**0.648**	**l-Isoleucine, trimethylsilyl ester**
7	12.118	0.056	Propanedioic acid, bis(trimethylsilyl) ester
8	**12.320**	**1.322**	**L-Valine, N-(trimethylsilyl)-, trimethylsilyl ester**
9	12.727	0.117	N,N-bis [2-Trimethylsiloxyethyl] ethanamine
10	13.685	7.051	Urea, N,N’-bis(trimethylsilyl)-
11	**14.012**	**0.520**	**L-Leucine, N-(trimethylsilyl)-, trimethylsilyl ester**
12	**14.173**	**26.110**	**Silanol, trimethyl-, phosphate (3:1)**
13	14.357	0.176	Diethylamine, 2,2’-bis(trimethylsiloxy)-
14	14.584	0.968	L-Isoleucine, N-(trimethylsilyl)-, trimethylsilyl ester
15	**14.647**	**0.657**	**l-Threonine, O-(trimethylsilyl)-, trimethylsilyl ester**
16	**14.834**	**0.790**	**Glycine, N,N-bis(trimethylsilyl)-, trimethylsilyl ester**
17	14.903	0.264	Methanamine, N,N-di(2-trimethylsilyloxyethyl)-
18	15.260	0.186	Butanedioic acid, bis(trimethylsilyl) ester
19	15.616	1.188	Propanoic acid, 2,3-bis[(trimethylsilyl)oxy]-, trimethylsilyl ester
20	**16.353**	**0.132**	**Serine tritms**
21	16.941	0.811	N,O,O-Tris(trimethylsilyl)-L-threonine
22	18.078	2.934	(R*,S*)-3,4-Dihydroxybutanoic acid triTMS
23	19.282	0.343	Malic acid, O-(trimethylsilyl)-, bis(trimethylsilyl)ester
24	19.495	0.091	Threitol, 1,2,3,4-tetrakis-O-(trimethylsilyl)-, D-
25	19.642	2.553	19.642 3,8-Dioxa-2,9-disiladecane, 2,2,9,9-tetramethyl-5, 6-bis[(trimethylsilyl)oxy]-, (R*,S*)-
26	**19.917**	**3.453**	**L-Aspartic acid, N-(trimethylsilyl)-**
27	20.350	1.452	L-Threonic acid, tris(trimethylsilyl) ether, trimethylsilyl ester
28	20.728	0.372	L-Threonic acid, tris(trimethylsilyl) ether, trimethylsilyl ester
29	20.948	0.430	3-Pyridinecarboxylic acid, 6-[(trimethylsilyl)oxy]-, trimethylsilyl ester
30	**21.895**	**3.125**	**Glutamine, tris(trimethylsilyl)-**
31	22.013	8.141	Amine, N,N,N-tris((trimethylsilyloxy)ethyl)-
32	22.237	0.278	Benzeneacetic acid, 4-[(trimethylsilyl)oxy]-, trimethylsilyl ester
33	22.347	0.172	1,3,5-Tris(trimethylsiloxy)benzene
34	22.600	0.339	3,4,5-Trihydroxypentanoic acid, tetrakis(trimethylsilyl)-
35	22.875	0.075	4H-1,3,5-Oxadiazine-4-carboxylic acid, 2- (dimethylamino)-6-(2-fluorophenyl)-4- (trifluoromethyl)-, ethyl ester
36	23.396	0.090	Arabinopyranose, tetrakis-O-(trimethylsilyl)-, à-D-
37	**24.934**	**3.193**	**L-Glutamic acid, N-acetyl-, bis(trimethylsilyl) derive**
38	25.268	0.177	9H-Purine, 9-(trimethylsilyl)-6-[(trimethylsilyl)oxy]-
39	25.455	6.844	1,2,3-Propanetricarboxylic acid, 2- [(trimethylsilyl)oxy]-, tris(trimethylsilyl) ester
40	29.170	0.252	Hexadecanoic acid, trimethylsilyl ester
41	31.677	0.210	9,12-Octadecadienoic acid (Z,Z)-, trimethylsilyl ester
42	35.325	0.530	Uridine, 2’,3’,5’-tris-O-(trimethylsilyl)-

**Table 2:** Chemical compounds identified in PPE via GC-MS analysis.

Peak Number	Retention Time	Area (%)	Identified Compounds
1	6.8061	0.5462	Cyclohexanone
2	7.1657	0.8791	2,5-Furandione, 3-methyl-
3	7.3045	1.2139	2,5-Furandione, 3-methyl-
4	7.5947	0.3992	2-Imino-4-methylpentanenitrile
5	7.9669	0.7547	2,4-Dihydroxy-2,5-dimethyl-3(2H)-furan-3-one
6	8.2634	0.0797	Allyl (3-methylbutyl) sulfide
7	8.4906	0.0928	Phenol
8	8.7113	0.1481	1-Cyclohexene-1-methanol
9	8.8817	0.6872	Oxirane, (butoxymethyl)-
10	9.3044	0.038	4-Oxopentyl formate
11	9.8027	0.1825	2-Methyl-3-vinyl-oxirane
12	10.0677	0.1256	2-Pentenoic acid, methyl ester
13	10.2885	0.5623	2,5-Dimethyl-4-hydroxy-3(2H)-furanone
14	10.7932	0.2125	6-Methyl-6-hepten-4-yn-3-ol
15	10.932	0.8212	Thymine
16	11.1275	0.5276	1,3-Cyclopentanedione, 2,2-dimethyl-
17	12.0108	0.0724	3(2H)-Furanone, dihydro-5-isopropyl-
18	12.2127	0.1121	N-(N-Allylformamide)ethyleneimine
19	12.3704	3.9396	4H-Pyran-4-one, 2,3-dihydro-3,5-dihydroxy-6-methyl-
20	13.405	0.1022	1,3,3-Trichloro-2-methyl-4-pentanone
21	13.6321	0.3758	4H-Pyran-4-one, 3,5-dihydroxy-2-methyl-
22	13.9034	0.3932	1,3,2-Oxazaborolane, 2-butyl-
23	14.0926	0.1731	Diethylvinylsilane
24	14.2314	0.1516	Hydroquinone
25	14.4396	0.248	Benzofuran, 2,3-dihydro-
26	14.6036	5.451	5-Hydroxymethylfurfural
27	15.3039	0.3331	5-Hydroxymethylfurfural
28	15.4742	0.423	1H-Pyrrole-2,5-dione
29	16.2123	0.0489	Ethanone, 1-(2-hydroxy-5-methylphenyl)-
30	16.5215	0.1349	4-Mercaptophenol
31	16.7549	0.2108	4-Mercaptophenol
32	17.1334	0.0698	2,5-Furandione, 3,4-dimethyl-
33	17.7769	0.0494	Methyl 2-thienylacetate
34	18.1238	0.4649	1-Ethyl-1-ethoxy-1-silacyclopentane
35	19.2909	0.4163	1,2,4-Benzenetriol
36	20.212	0.5949	Sucrose
37	20.3193	0.407	Sulfurous acid, decyl 2-propyl ester
38	20.906	0.1494	Heptanoic acid
39	20.9753	0.1092	.alpha.-D-Glucopyranose, 4-O-.beta.-D-galactopyranosyl-
40	22.01	0.1773	Sucrose
41	22.6282	0.1907	1,5-Anhydro-d-mannitol
42	23.6691	0.9486	Methyl-4-azido-4-desoxy.beta.l-arabinopyranoside
43	23.7322	1.8149	Polygalitol
44	24.3189	0.1601	d-Mannitol, 1,4-anhydro-
45	24.4766	0.1939	2-O-Mesyl arabinose
46	24.6091	0.3645	3-Deoxy-d-mannonic acid
47	27.6057	0.1994	1,2-Cyclooctanedione
48	28.5899	0.0721	n-Hexadecanoic acid
49	32.7851	0.1273	Hexatriacontyl pentafluoropropionate
50	33.2898	0.1952	Hexatriacontyl pentafluoropropionate
51	33.416	0.0933	Octacosyl trifluoroacetate
52	33.7314	0.3113	Sulfurous acid, octadecyl 2-propyl ester
53	34.1352	0.468	Oxalic acid, isobutyl hexadecyl ester
54	34.2803	0.2256	1-Hexacosene
55	34.9301	2.2523	Hexatriacontyl pentafluoropropionate
56	35.2392	0.3489	Oxalic acid, cyclobutyl pentadecyl ester
57	35.6366	1.3339	Oxalic acid, cyclobutyl heptadecyl ester
58	35.9079	0.3096	Tetracosyl heptafluorobutyrate
59	36.0341	0.3319	Oxalic acid, cyclobutyl heptadecyl ester
60	36.2422	1.0202	Oxalic acid, cyclobutyl heptadecyl ester
61	36.6208	1.2003	Oxalic acid, 6-ethyloct-3-yl ethyl ester
62	37.6617	1.1687	Heneicosane, 11-(1-ethylpropyl)-
63	38.0339	2.4216	Ethanol, 2-(octadecyloxy)-
64	38.4818	1.93	Octatriacontyl pentafluoropropionate
65	38.8856	3.6393	Oxalic acid, allyl octadecyl ester
66	39.548	2.7195	Octatriacontyl pentafluoropropionate
67	40.57	5.5135	Pentatriacontane
68	40.8539	10.9949	Pentatriacontane
69	41.2702	2.1909	Nonadecane
70	42.513	12.6999	Heptadecane, 9-octyl-
71	43.0619	2.0224	Dotriacontyl pentafluoropropionate
72	43.4404	1.807	17-Pentatriacontene
73	44.065	0.8617	Oxalic acid, cyclobutyl hexadecyl ester
74	44.475	2.504	Ethanol, 2-(octadecyloxy)-
75	44.822	1.2853	Dotriacontyl heptafluorobutyrate
76	45.2194	1.6025	Oxirane, [(hexadecyloxy)methyl]-
77	45.5349	1.0744	Tetratriacontyl pentafluoropropionate
78	46.1973	0.8266	Hexacosyl heptafluorobutyrate
79	47.5536	9.5266	Heneicosane
80	48.3233	1.1708	Cyclohexane, 1,1’-(2-methyl-1,3-propanediyl)bis-
1	6.8061	0.5462	Cyclohexanone
2	7.1657	0.8791	2,5-Furandione, 3-methyl-
3	7.3045	1.2139	2,5-Furandione, 3-methyl-
4	7.5947	0.3992	2-Imino-4-methylpentanenitrile
5	7.9669	0.7547	2,4-Dihydroxy-2,5-dimethyl-3(2H)-furan-3-one
6	8.2634	0.0797	Allyl (3-methylbutyl) sulfide
7	8.4906	0.0928	Phenol
8	8.7113	0.1481	1-Cyclohexene-1-methanol

**Table 3:** T_gel_ of thermoresponsive gels with different concentrations of PEG 400 and/or starch, and loading with HLRE and PPE, n = 3.

Formulation	PEG 400 (% *w*/*w*)	Starch (mg/L)	PPE (mg/mL)	HLRE (mg/mL)	T_gel_ (°C)
F1 (blank)	-	-	-		24.3 ± 0.6
F2	5	-	-		27.3 ± 0.6
F3	10	-	-		30.0 ± 1.0
F4	15	-	-		33.3 ± 1.2
**F5**	**22**	-	-		**37.0 ± 1.0**
F6	-	500	-		22.3 ± 0.6
F7	-	1000	-		23.7 ± 0.6
F8	-	1500	-		24.3 ± 0.6
F9	1	1500	-		21.0 ± 1.7
F10	5	1500	-		24.3 ± 0.6
F11	10	1500	-		24.7 ± 0.6
F12	15	1500	-		24.3 ± 0.6
F13	20	1500	-		17.3 ± 3.1
F14	22	1500	-		46.7 ± 1.5
**F15**	**22**	**-**	**1.00**	**0.125**	**26.7 ± 1.2**
**F16**	**22**	**-**	**0.50**	**0.125**	**26.2 ± 1.3**
**F17**	**22**	**-**	**0.25**	**0.125**	**26.2 ± 0.8**
**F18**	**22**	**-**	**0.125**	**0.125**	**25.7 ± 0.3**
**F19**	**22**	**-**	**0.06**	**0.125**	**25.8 ± 0.3**

**Table 4:** Zone of inhibition (mm) against *Staphylococcus aureus* for PPE, n = 3.

Sample (mg/mL)		Zone of Inhibition (mm)
PPE	1	10.1 ± 1.2
	0.5	8.00 ± 0.0
	0.25	-
	0.125	-
	0.06	-
F15–F19		-
Gentamicin (Positive control)		29.7 ± 0.6

F15–F19—gels containing HLRE and different concentrations of PPE.

## Data Availability

Not applicable.

## Supplementary Materials

The following supporting information can be downloaded at: https://www.mdpi.com/article/10.3390/gels9010048/s1, Figure S1: Cytotoxicity effect of thermoresponsive gels containing HLRE in normal human dermal fibroblasts (NHDF) as determined by AlamarBlue assay at 24 h of incubation, n = 3; Figure S2. GC-MS analysis of HLRE (A) and PPE (B).
