# Supplementary material for: Potential Hemostatic and Wound Healing Effects of Thermoresponsive Wound Dressing Gel Loaded with Lignosus rhinocerotis and Punica granatum Extracts
Source: Gels. 2023 Jan 6;9(1):48. doi: 10.3390/gels9010048 (PMC9858555; doi:10.3390/gels9010048)
Supplement: Supplementary file 1 [file gels-09-00048-s001.zip › gels-2102956-supplementary.pdf]

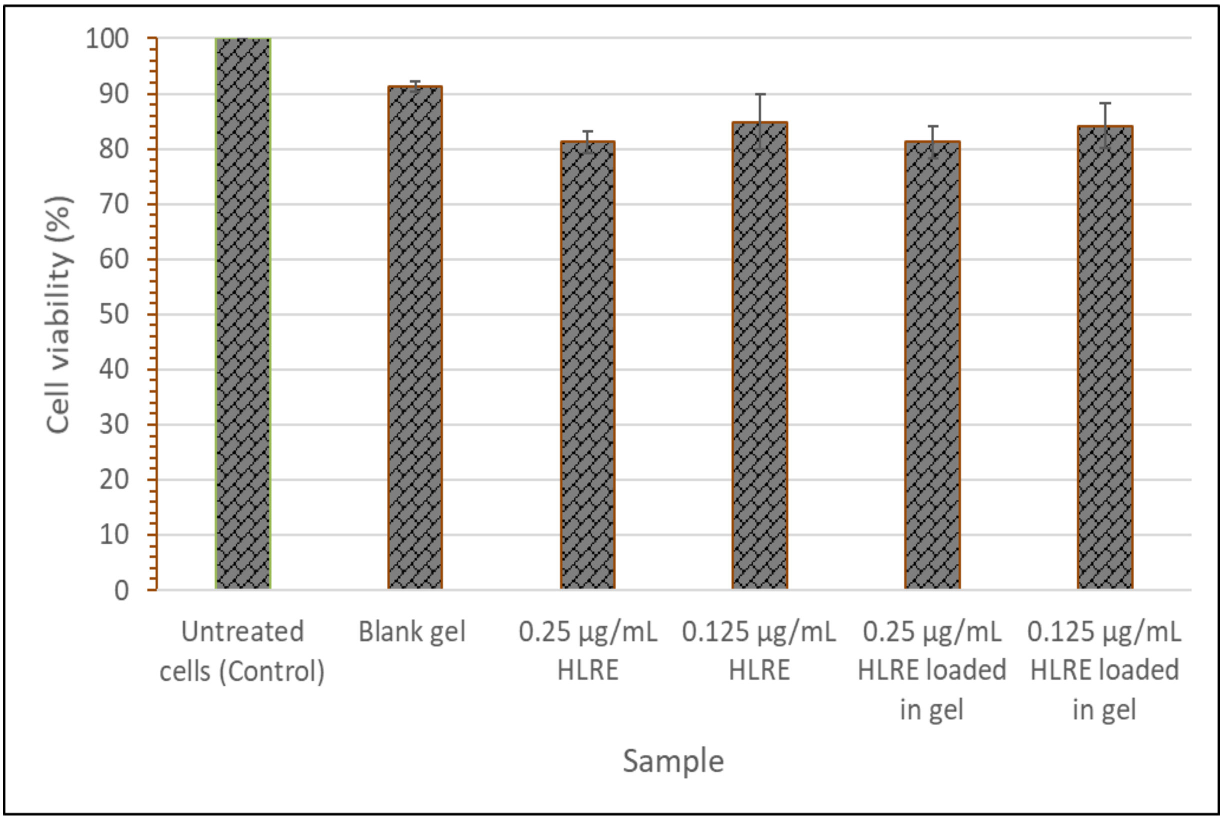

Figure S1. Cytotoxicity effect of thermoresponsive gels containing HLRE in normal human dermal fibroblasts (NHDF) as determined by AlamarBlue assay at 24 h of incubation, n=3.

Footnote: Blank gel indicates unloaded thermoresponsive gels made of PF127 and PEG (F5); HLRE indicates hot extract of L. rhinocerotis

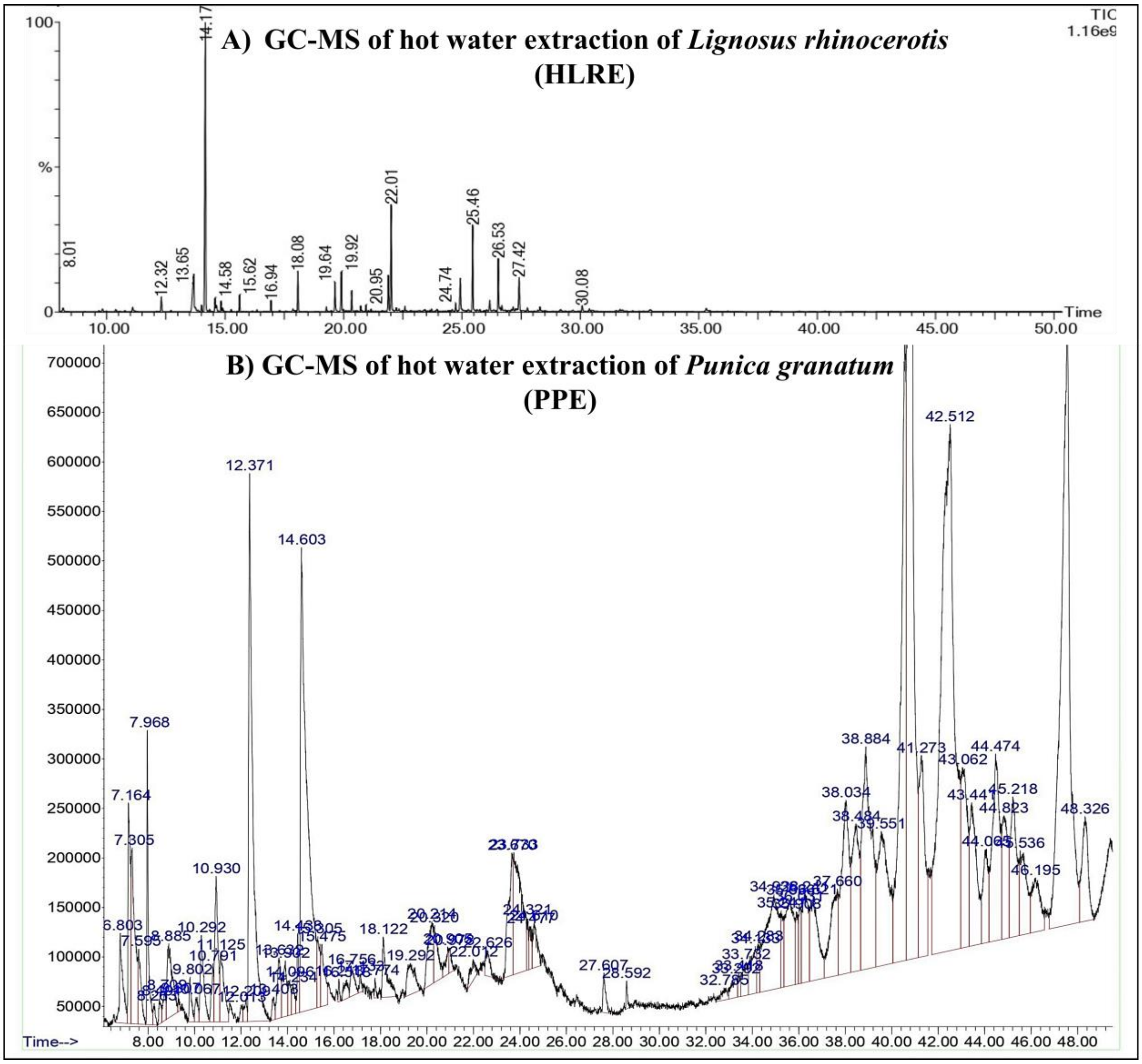

Figure S2. GC-MS analysis of HLRE **(A)** and PPE **(B)** .
